# Cytokine Networks in Alcohol Use Disorder: A Narrative Review Highlighting Research Gaps and Future Priorities

**DOI:** 10.3390/medsci14020179

**Published:** 2026-04-02

**Authors:** Carmen M. Galvez-Sánchez, Julio A. Camacho-Ruiz, Cristina González-Lara, Rosa M. Limiñana-Gras

**Affiliations:** 1 Department of Personality, Evaluation and Psychological Treatment, Faculty of Psychology and Speech Therapy, University of Murcia, Building 31, 30100 Murcia, Spain; liminana@um.es; 2 Regional International Campus of Excellence (CEIR) Mare Nostrum Campus (CMN), 30100 Murcia, Spain; 3 Foundation Project Man Jaén, 23002 Jaén, Spain; 4 Department of Physical Therapy, Faculty of Health Sciences, University of Jaén, 23071 Jaén, Spain; cgl00038@red.ujaen.es; 5 Assisted Reproduction Unit, QuironSalud Murcia Medical Center, 30008 Murcia, Spain

**Keywords:** Alcohol Use Disorder (AUD), cytokines, interleukins (ILs), neuroinflammation, biomarkers

## Abstract

**Background/Objectives**: Alcohol Use Disorder (AUD) represents a significant global public health challenge that is associated with cognitive deficits, immune dysfunction, and increased susceptibility to different comorbidities. Recent evidence suggests that neuroimmune signalling, particularly microglial activation and cytokine-mediated pathways, plays a critical role in the development, persistence, and relapse vulnerability of AUD. This narrative review aims to synthesize current evidence on the role of cytokines and interleukins (ILs) in AUD, emphasizing their modulation during alcohol exposure, withdrawal, and abstinence. **Methods**: A comprehensive narrative review methodology was employed, including a search in PubMed, Scopus, and Web of Science using relevant keywords. Peer-reviewed studies published in English that examined cytokine and interleukin profiles in adults with AUD were included. The main findings were synthesized into thematic domains to identify recurring patterns, inconsistencies, and research gaps. **Results**: AUD is associated with significant alterations in cytokine profiles. Pro-inflammatory markers such as IL-1β, IL-6, tumour necrosis factor alpha (TNF-α), IL-8, and IL-18 are elevated during active alcohol use and early abstinence, while anti-inflammatory markers like IL-10 show fluctuations. These immune changes are linked to systemic inflammation, neurotoxicity, and AUD severity. Cytokine levels tend to normalize with sustained abstinence, although severe AUD may lead to prolonged immune dysregulation. Associations between inflammatory markers and psychiatric symptoms, including anxiety and depression, were also observed. **Conclusions**: Immune dysregulation plays a central role in AUD pathophysiology, with cytokines serving as potential biomarkers for disease progression and treatment response. Future research should focus on longitudinal studies, diverse patient populations, and mechanistic investigations to refine biomarker utility and develop targeted immunomodulatory therapies. Addressing inflammation and neuroplasticity may enhance clinical outcomes in AUD management.

## 1. Introduction

Alcohol Use Disorder (AUD) represents a major global public health and social challenge that is associated with substantial morbidity and mortality [1,2]. Chronic and excessive alcohol consumption can result in both reversible and irreversible cognitive deficits and is linked to structural alterations in the brain [3,4,5]. Moreover, alcohol abuse may also affect immune function [6,7] and increase susceptibility to several malignancies (e.g., cancers of the colon, upper aerodigestive tract, liver, and breast, etc.) [2,8].

Recent work has increasingly focused on the neurobiological underpinnings of AUD, and converging evidence suggests that neuroimmune signalling—particularly microglial activation and cytokine-mediated pathways—may contribute to both the development and persistence of the disorder, including vulnerability to relapse [9,10,11]. Cytokines comprise a heterogeneous group of proteins and glycoproteins produced by multiple cell types that function primarily as key regulators of immune and inflammatory responses. In addition, they act as growth and differentiation factors for a range of cells, particularly within the hematopoietic lineage. Their central role is to mediate intercellular communication, thereby modulating the activity of a broad spectrum of target cells. Cytokines can either induce or suppress the synthesis of other cytokines and their receptors, promoting synergistic interactions or, alternatively, exerting antagonistic effects. They are also characterized by pleiotropy, reflecting their capacity to act across diverse tissues and elicit multiple biological effects [12]. In parallel, findings from immunopsychiatry indicate that immune and inflammatory signalling in those with severe mental illnesses differ from that of healthy controls and may vary by illness stage and symptom dimensions, highlighting substantial biological heterogeneity within diagnostic categories [9,10,11].

In addition, several immunological parameters—including α TNF-α and interleukins (ILs)-1β, IL-8, IL-12, and IL-13—appear to be elevated in individuals with alcohol abuse or dependence. In contrast, IL-6 concentrations usually decrease during detoxification treatment in alcohol-dependent patients without liver disease [12,13,14,15].

Immune alterations associated with alcohol use are multifactorial and vary by exposure pattern, host comorbidities, and the specific immune pathways assessed [16,17]. Alcohol can activate innate immunity both centrally and peripherally. In the brain, ethanol-related neuroimmune signalling is closely linked to microglial activation and downstream proinflammatory cascades implicated in neurotoxicity and AUD pathophysiology [18,19]. Systemically, alcohol-induced dysbiosis and increased intestinal permeability facilitate translocation of microbial products such as lipopolysaccharide (LPS) into the portal circulation, amplifying hepatic inflammation and cytokine production that can propagate inflammatory signalling to the central nervous system via the bloodstream [20,21]. Moreover, acute and chronic alcohol exposure can exert divergent, time-dependent immunomodulatory effects. In fact, acute intoxication may show a biphasic response with early proinflammatory changes followed by later anti-inflammatory or immunosuppressive features, whereas sustained heavy use is more consistently associated with persistent immune dysregulation and proinflammatory tone [16,22]. Characterizing these dynamic, context-dependent immune signatures may help clarify mechanisms underlying AUD onset, maintenance, and progression, and inform targeted intervention strategies [16,17].

Unlike prior research that primarily relies on cross-sectional data, this narrative review emphasizes the relevance of temporal variability in cytokine profiles, highlighting how inflammatory markers fluctuate based on drinking patterns, recency of alcohol exposure, and the progression of AUD. This approach may help contextualize immunological findings within clinically meaningful phases of the disorder, such as detoxification and early recovery, and identify priorities for future mechanistic research.

Based on the growing body of scientific evidence linking immune dysregulation to the pathophysiology of AUD, this review aims to provide a comprehensive synthesis of current findings while addressing critical gaps in the literature. The following section outlines the specific research questions and objectives that guided this investigation, providing a structured framework for exploring the complex relationship between immune signalling and AUD.

### Clarification of Research Questions and Study Objectives 

This narrative review aims to address the following research questions:

– What is the role of cytokine and interleukin networks in the pathophysiology of AUD?
– Specifically, how do alcohol consumption and abstinence modulate pro-inflammatory and anti-inflammatory signalling?
– What are the mechanistic pathways linking immune dysregulation to AUD-related neurobiology and clinical phenotypes?
– What are the sources of heterogeneity in immune responses among individuals with AUD?
– What are the translational implications of immune dysregulation in AUD?
– Can cytokines and ILs serve as biomarkers for disease progression, treatment response, or risk stratification in AUD?

The primary objectives of this review are as follows:

1. To synthesize and critically appraise current evidence on the role of cytokines and ILs in AUD.
2. To identify dynamic immune signatures that vary across different stages of AUD, including active drinking, withdrawal, and abstinence.
3. To highlight potential biomarkers and immunomodulatory therapeutic strategies that could improve clinical outcomes in AUD.
4. To identify research gaps and propose future priorities for advancing the understanding of immune dysregulation in AUD.

## 2. Review Approach

This manuscript employs a comprehensive narrative review methodology to present a conceptually oriented synthesis of the literature on cytokine and interleukin networks in AUD, thereby enabling interpretative integration across a diverse and methodologically heterogeneous evidence base and facilitating the identification of research gaps and future priorities. A narrative approach was considered appropriate because the available evidence is methodologically heterogeneous, including studies that differ substantially in design, sample characteristics, clinical stage of AUD, biological markers assessed, and analytical procedures.

The search was conducted in PubMed, Scopus, and Web of Science, which were selected because together they provide broad coverage of biomedical, clinical, and interdisciplinary research relevant to AUD, inflammation, and neuroimmune mechanisms. The search covered studies published up to 2026 using combinations of the following terms and related keywords: “Alcohol Use Disorder,” “AUD,” “cytokines,” “interleukins,” “inflammation,” “immune dysregulation,” and “neuroinflammation,” with Boolean operators applied. Eligible studies were peer-reviewed empirical articles published in English that examined cytokine and/or interleukin profiles, signalling pathways, or inflammatory networks in adults with AUD or clinically relevant alcohol exposure, including comparisons across drinking states (e.g., active use, withdrawal, detoxification, and abstinence). Studies were excluded if they were duplicates. To maintain a focused synthesis of the empirical and scholarly literature, grey literature was not included. Given the narrative nature of the review, the aim was not to perform a formal systematic selection or quantitative appraisal, but rather to identify relevant literature and synthesize it through thematic summarizing, paraphrasing, and interpretative comparison across studies. Therefore, the main results from included studies were synthesized into thematic domains, using descriptive and comparative approaches to map recurring patterns, sources of inconsistency, main limitations, and priorities for future research.

## 3. Results

The results of this narrative review are organized into thematic domains to provide a structured and comprehensive synthesis of the evidence. First, the review examines the associations between cytokine and interleukin networks and AUD, highlighting key inflammatory markers and their roles in the pathophysiology of the disorder. Second, it explores the dynamic changes in cytokine levels across different stages of AUD, including withdrawal and abstinence, to elucidate the temporal variability of immune dysregulation. Third, the review investigates the correlations between clinical severity, affective symptoms, and inflammatory markers, emphasizing the interplay between immune activation and neuropsychological outcomes. Finally, the limitations of existing studies and future research priorities are discussed to address gaps in the literature and propose directions for advancing in this field.

### 3.1. Cytokine and Interleukin Networks in Individuals with Alcohol Use Disorder (AUD)

Several studies have documented associations between inflammatory dysregulation and AUD [23,24,25,26,27,28,29,30]. In this regard, a history of alcohol use was associated with significant differences across all assessed cytokines. Specifically, the alcohol group exhibited higher plasma concentrations of IL-1β, IL-6, and TNF-α compared with the control group. In contrast, plasma concentrations of IL-4, IL-17A, and IFN-γ were lower in the alcohol group than in controls [23,24,25].

In the same line, Balan et al. [26] reported that IL-1β levels are significantly elevated in high-risk individuals. Specifically, IL-1β—together with other immunological mediators such as IL-18, IL-7, and CCL11—was markedly increased among participants with Alcohol Use Disorders Identification Test (AUDIT) scores ≥ 6, consistent with potential immune dysregulation and a pro-inflammatory profile associated with alcohol consumption.

García-Calvo et al. [27] also found that inflammatory cytokines, particularly IL-6, have been associated with monocyte activation and systemic inflammation in patients with AUD. Elevated IL-6 levels correlated with clinical and laboratory characteristics, including older age and higher levels of AST, GGT, and ferritin, a higher mean corpuscular volume (MCV), an erythrocyte sedimentation rate (ESR) > 20 mm, and C-reactive protein (CRP) > 5 mg/L. In addition, IL-6 levels showed negative associations with alcohol consumption, total cholesterol, and albumin, which may indicate a state of systemic inflammation and early-stage liver injury.

Moreover, Tyler et al. [28] analysed cytokines and ILs in relation to immune dysregulation in AUD. The authors reported that alcohol exposure appears to perturb immune function and is associated with measurable shifts in circulating inflammatory mediators. Acute alcohol intake has been reported to decrease TNF-α while increasing IL-6. In contrast, chronic alcohol use is linked to more persistent immunological alterations, including elevated concentrations of proinflammatory markers such as TNF-α, IL-6, IL-8, and IL-18, alongside changes in anti-inflammatory mediators including IL-10 and interleukin-1 receptor antagonist (IL-1RA). In addition, correlation analyses further supported the clinical relevance of inflammatory signalling. Proinflammatory markers, including IL-18 and TNF-α, were positively associated with AUD severity and with measures of anxiety and depressive symptoms, indicating that greater inflammatory activation may track with both disorder severity and comorbid affective burden. Conversely, IL-8 was negatively correlated with anxiety/depression measures within the abstinent AUD group, suggesting that the relationship between inflammation and psychiatric symptoms may vary by drinking status and phase of recovery.

Grodin et al. [24] also highlighted the role of ILs—including IL-6, IL-8—and TNF-α and CPR, which are consistently elevated in individuals with AUD compared with controls. These proinflammatory cytokines have been associated with alcohol consumption and AUD severity, suggesting that increased alcohol intake may modulate the immune system. Moreover, the study specifically identifies elevated IL-8 levels in individuals with AUD who exhibit clinically significant insomnia symptoms, a pattern not observed for other inflammatory markers such as IL-6, TNF-α, or CRP. IL-8, a proinflammatory chemokine, appears to be linked to sleep quality and insomnia symptoms in people with AUD. The authors also note that IL-8 production in peripheral blood mononuclear cells is associated with specific inflammatory signalling pathways, such as the activator protein 1 (AP-1) transcription factor pathway, which has been related to alcohol consumption and craving.

Nikou et al. [29] reported that across the detoxification period, IL-7 concentrations were elevated in all patients. Levels remained consistently high during the first two weeks following initiation of detoxification, after which, a decline was observed; however, IL-7 concentrations remained significantly higher than those of the control group at the end of treatment. IL-10 concentrations were significantly lower than in controls at admission and exhibited a linear increase throughout detoxification; nevertheless, values remained slightly lower than those of the control group at the end of therapy. In contrast, G-CSF concentrations were significantly elevated on admission and then decreased linearly over the course of detoxification, reaching near-normal values by the end of treatment. In this study, the detoxification protocol comprised an initial 10 mg intramuscular injection of diazepam at admission, followed by oral diazepam 10 mg administered four times daily. From day 4 onward, the dose was progressively tapered to 5 mg orally, and diazepam was discontinued between days 12 and 14. Throughout hospitalization, patients received vitamins B1, B6, B12, A, E, and C, as well as folate [29]. Table 1 provides a comprehensive overview of some of the most relevant inflammatory markers associated with AUD, highlighting their roles in immune dysregulation and their potential implications for disease progression and treatment.

### 3.2. Changes in Cytokine Levels Across Different Stages of Alcohol Use Disorder (AUD)

Cytokine levels may vary across different stages of the clinical course of AUD [23,27,28,30]. In abstinent patients with AUD, different alterations in plasma cytokine concentrations were observed relative to control subjects. Specifically, IL-1β, IL-6, and TNF-α were increased, whereas IL-4, IL-17A, and interferon gamma (IFN-γ) were reduced. These findings may indicate that abstinent individuals with AUD exhibit a disrupted plasma cytokine profile that may be associated with inflammation related to chronic alcohol exposure. In addition, plasma IL-6 and IL-17A concentrations were associated with the presence of comorbid hepatic and pancreatic diseases [23].

Similarly, Tyler et al. [28] reported that IL-8 levels remained elevated in the abstinent AUD group (AB) compared to healthy controls and currently drinking individuals. This suggests a persistent proinflammatory state in individuals with severe AUD, even after 6+ weeks of abstinence.

In addition, the study of García-Calvo et al. [27] pointed out that prior studies have reported reductions in biomarkers of inflammation and intestinal permeability (such as sCD14 and IL-6) after approximately 2–3 weeks of alcohol abstinence. This suggests that abstinence may beneficially attenuate systemic inflammation and monocyte activation in patients with AUD. However, in their research, biomarkers were only assessed once—two days after admission—so changes in these markers following a longer period of abstinence were not evaluated.

**Figure 1:**
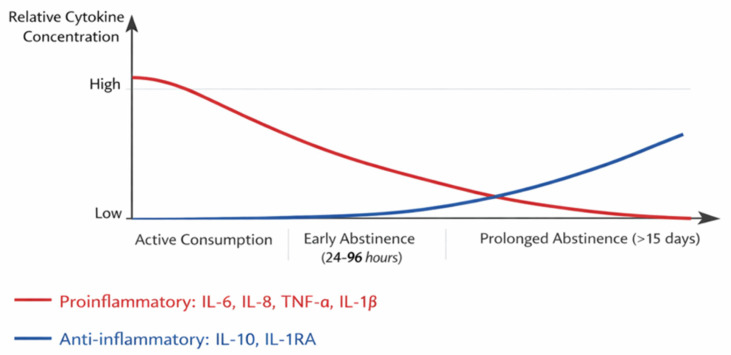
Changes in Cytokine Levels Across the Stages of Alcohol Use Disorder (AUD). **Note:** Figure developed by authors from the data reported in the analysed studies. Systemic inflammation has been reported during active alcohol consumption and early abstinence. Prolonged abstinence appears to be associated with lower levels of pro-inflammatory cytokines and higher levels of anti-inflammatory cytokines. In severe AUD, IL-8 may remain elevated after weeks of abstinence.

To visually represent the dynamic fluctuations in cytokine levels during the different stages of AUD, a diagram has been included below. Figure 1 illustrates the relative concentrations of pro-inflammatory cytokines (IL-6, IL-8, TNF-α, IL-1β) and anti-inflammatory cytokines (IL-10, IL-1RA) across three key phases: active alcohol consumption, early abstinence (24–96 h post-cessation), and prolonged abstinence (beyond 15 days). The diagram highlights the systemic inflammatory state observed during active drinking and early abstinence, characterized by elevated pro-inflammatory markers and suppressed anti-inflammatory mediators. It also may reflect the gradual normalization of cytokine levels during prolonged abstinence, emphasizing the potential recovery of immune function. This visual aid complements the textual analysis and provides a clearer understanding of the immune dysregulation associated with AUD, offering insights into its clinical implications and potential therapeutic targets.

### 3.3. Associations of Clinical Severity and Affective Symptoms with Inflammatory and Neurotrophic Markers in Individuals with Alcohol Use Disorder (AUD)

It has been reported that cytokines have been associated with mood states in patients with psychiatric disorders. For example, elevated levels of inflammatory cytokines such as IL-6 and TNF-α have been associated with depression. In addition, positive associations have been observed between anxiety (e.g., panic disorder) and the cytokines IL-6, IL-1β, and IL-5. It has also been described that, in patients with AUD, IL-6 is positively associated with depression and IL-10 is negatively associated with anxiety [23].

Similarly, Balan et al. [26] reported a significant association between alcohol craving in individuals with AUD and intestinal dysfunction, alongside elevations in pro-inflammatory biomarkers such as IL-1β and TNF-α. This finding reinforces the proposed relationship between immune activity and clinically relevant symptoms of AUD. Regarding neurotrophic markers, the study measured brain-derived neurotrophic factor (BDNF) but did not detect significant differences between high- and low-risk alcohol consumption groups. No meaningful associations were observed between BDNF levels and AUDIT or Alcohol Use Disorders Identification Test–Consumption (AUDIT-C) scores. Nevertheless, the authors suggest that future research incorporating anti-inflammatory mediators such as IL-1RA or IL-10 could provide a more balanced characterization of immune regulation in AUD.

García-Calvo et al. [27] also reported elevated levels of IL-6 in individuals with AUD compared to healthy controls. IL-6 has been associated with anxiety and depressive symptoms during alcohol withdrawal, although findings are inconsistent. IL-6 levels have also been linked to comorbid depressive disorders in individuals with AUD. Regarding other psychiatric disorders, higher blood IL-6 levels have been observed in other psychiatric conditions, such as post-traumatic stress disorder, major depression, and schizophrenia. However, only a few studies on AUD explicitly excluded individuals with other psychiatric disorders, which could act as confounding factors. Overall, while IL-6 appears to play a role in mood symptoms associated with AUD, further research is needed to clarify its relationship with anxiety and depression.

In the same line, Tyler et al. [28] reported significant associations between clinical severity and biomarker profiles in AUD. Specifically, greater AUD severity and higher anxiety/depression scores were positively correlated with the proinflammatory cytokines IL-18 and TNF-α, whereas both clinical measures were negatively correlated with BDNF. IL-18 is an immune-activating cytokine implicated in chronic inflammation and immune dysregulation, and its positive association with AUD severity and anxiety/depression is presented as consistent with evidence of altered IL-18 signalling in stress- and addiction-relevant neurocircuitry in animal models, as well as increased IL-18 expression in peripheral immune cells in alcohol-related liver disease. Similarly, TNF-α—widely characterized as a key mediator of systemic inflammation—was positively correlated with AUD severity and with anxiety/depression, consistent with the view that AUD is associated with a heightened inflammatory tone and may be linked to neuroinflammation and mood symptoms. In contrast, BDNF, described as central to synaptic plasticity and overall brain health and noted to have anti-inflammatory effects, was inversely correlated with AUD severity and anxiety/depression, supporting the interpretation that more severe clinical presentation is accompanied by lower neurotrophic support. Overall, these correlation patterns are interpreted as reflecting a coordinated relationship between inflammatory activation (IL-18, TNF-α), reduced neuroplasticity-related signalling (BDNF), and the clinical expression of AUD and comorbid affective symptoms.

Bramness et al. [25] also reported that elevated levels of the cytokine IL-6 have been associated with suicide attempts in patients with AUD. Although no statistically significant differences in IL-6 levels were observed between patients with and without suicide attempts in the bivariate analysis, the multivariable analysis—adjusted for factors such as sex, nicotine use, somatic comorbidities, and the use of anti-inflammatory medications—showed a significant association between higher IL-6 levels and suicide attempts (*p* = 0.033). These findings support prior evidence suggesting that IL-6 may represent a biological marker related to suicidal behaviour. However, the authors caution that the clinical applicability of IL-6 as an individual-level predictive biomarker remains limited.

Finally, Grodin et al. [24] reported that, among individuals with AUD, those with clinically significant insomnia symptoms showed higher IL-8 levels than those without insomnia or with subclinical insomnia, whereas no significant differences were found for IL-6, TNF-α or CRP. With regard to sex- and AUD severity-related interactions, men with AUD and clinical insomnia had higher IL-8 levels than women and the other comparison groups. Similarly, individuals with severe AUD and clinical insomnia presented higher IL-8 concentrations than those with moderate AUD or without insomnia. Overall, these findings suggest that insomnia in the context of AUD is associated with systemic inflammation, with IL-8 emerging as a potential biomarker in this relationship. Sex and AUD severity may further influence IL-8 levels, indicating greater vulnerability in men and in individuals with more severe AUD. Further research is warranted to clarify these findings.

### 3.4. Main Limitations and Future Lines of Research

The reviewed studies on cytokine and interleukin networks in individuals with AUD present limitations that may undermine the robustness and generalizability of their findings. Sample sizes are frequently small, reducing statistical power, and many investigations rely on cross-sectional designs, precluding evaluations of within-subject changes in cytokine profiles during detoxification to sustained abstinence. Generalizability is further constrained by selective sampling strategies that exclude clinically relevant subgroups (e.g., currently drinking individuals; patients with severe liver or pancreatic disease; or those with psychiatric comorbidities). Across studies, inconsistent findings likely reflect substantial clinical heterogeneity—particularly differences in recency of alcohol intake, dependence severity, and comorbid conditions (including alcoholic liver disease, alcoholic hepatitis, and concurrent infections such as hepatitis B or C) that can amplify pro-inflammatory signalling.

Methodological variability also complicates comparisons, including differences in laboratory platforms, as well as variation in sample collection timing, storage, and processing, all of which may affect measurement accuracy and standardization. Finally, the limited use of standardized clinical instruments (e.g., the Clinical Institute Withdrawal Assessment for Alcohol) reduces the capacity to characterize withdrawal severity and objectively relate detoxification outcomes to inflammatory markers.

Future research should prioritize larger and more diverse cohorts, longitudinal designs, and standardized clinical and laboratory protocols to improve reliability and clinical interpretability. Table 2 summarizes the main limitations identified in the reviewed studies, emphasizing methodological constraints and areas for future research to enhance the understanding of immune dysregulation in individuals with AUD.

Moreover, across the included studies, limitations and proposed future directions consistently pointed to the need for longitudinal designs with longer follow-up to track post-detoxification and abstinence-related changes in cytokines and related biomarkers (e.g., CD163, sCD14, IL-6), alongside objective clinical documentation of detoxification outcomes using standardized instruments such as CIWA-Ar. The literature also emphasized broader and more representative sampling—larger cohorts; greater inclusion of women with consideration of menopausal/hormonal factors; recruitment of individuals with active alcohol use, milder drinking phenotypes, and psychiatric patients without substance-use histories; and targeted cohorts with comorbid conditions, particularly established liver disease and severe hepatic/pancreatic involvement.

Methodologically, authors recommended expanding immune panels and evaluating candidate biomarkers (e.g., IL-7, IL-10, G-CSF, and IL-6) for diagnostic, staging, monitoring, and prognostic purposes, while advancing mechanistic work on cytokine interactions, immune-cell dysfunction, monocyte activation, metabolic disturbances, transcriptional regulation (e.g., STAT3), and alcohol-related autoimmune processes. Additional priorities included assessing the impact of detoxification medications (e.g., diazepam, vitamin supplementation), comparing detoxification protocols, examining how targeted interventions (e.g., HCV treatment, metabolic management, sustained abstinence) influence biomarker trajectories and outcomes, and exploring AUD–comorbidity interactions to inform preventive approaches and the clinical use of immunological parameters as diagnostic tools and biomarkers, including in psychiatric comorbidity.

In addition, the clinical implementation of biomarkers also entails several challenges. Ensuring validity and reproducibility is essential, such that biomarkers yield consistent and replicable results across different populations and clinical contexts. In this regard, variability in analytical methods, sample storage procedures, and study conditions can compromise measurement precision and, consequently, the reliability of findings.

A further barrier is the limited standardization of methods. The absence of standardized protocols for biomarker assessment can hinder clinical uptake, underscoring the need for uniform procedures for sample collection, processing, and analysis. In parallel, costs and accessibility remain important concerns, as some biomarkers require advanced and expensive technologies for detection, thereby restricting their use in resource-limited clinical settings.

Interpreting biomarker results can also be complex, particularly because biomarker levels may be influenced by multiple factors, including comorbidities, medications, age, sex, and lifestyle. This complexity is compounded by the relative scarcity of longitudinal studies, which are needed to clarify how biomarker levels change over time and how these trajectories relate to disease progression and treatment response.

Regulatory and approval requirements represent another substantive obstacle, as biomarkers must meet stringent regulatory standards before being authorized for clinical use, a process that can be lengthy and costly. Beyond regulation, clinical acceptance is critical: healthcare professionals require training in biomarker utilization and interpretation, and clinical utility must be clearly demonstrated for biomarkers to be adopted in routine practice. Finally, successful integration into healthcare systems depends on adequate infrastructure, including specialized laboratories and robust data-management systems, which may be challenging to establish in certain settings.

Importantly, the current literature remains predominantly correlational and does not have firm conclusions on whether peripheral cytokine alterations are causal drivers of AUD-related behavioral and affective changes, or what the downstream consequences of alcohol exposure and withdrawal or compensatory adaptive responses are. This limitation is especially relevant because many cytokines and chemokines exert pleiotropic and state-dependent effects, making simplistic interpretations inappropriate. Future research should therefore prioritize mechanistic and longitudinal designs with repeated within-subject assessments, precise characterization of alcohol exposure and withdrawal timing, standardized biospecimen collection, and experimental approaches capable of testing whether modulation of peripheral inflammatory signalling is sufficient to influence clinically relevant outcomes.

Addressing these challenges is essential to ensuring that biomarkers can be used effectively and reliably in clinical practice, thereby improving the diagnosis, monitoring, and treatment of conditions such as AUD.

## 4. Discussion

The immune system has been reported to regulate behaviour through inflammatory signalling within the central nervous system (CNS), and several studies have shown that the pharmacodynamic effects of alcohol and other substances involve central modulation of immunological signalling pathways [31,32]. Within this network, cytokines are key mediators of cellular communication and activation, regulating inflammatory processes, cell migration, and proliferation [33]. They may access the brain via the blood–brain barrier or be produced locally by non-neuronal cells, including astrocytes and microglia [34].

Current evidence further suggests that alcohol directly modulates immune function and promotes neuroinflammation by altering inflammatory mediators both centrally and peripherally [35,36]. Alcohol consumption has been proposed to disrupt cytokine-mediated microglia–neuron interactions [37], although this interpretation has been challenged by in vivo human neuroimaging studies [38,39], and further research is needed to clarify these effects in adult patients with AUD [28]. Because inflammatory cytokine expression profiles change during the development of AUD and appear to normalize following cessation, this normalization may contribute to clinical improvement [40].

Neuroimmune signalling may therefore play an important role in both the development and progression of AUD [41]. Chronic heavy alcohol consumption can alter the adaptive immune profile [12], activate toll-like receptor signalling, increase intestinal permeability, and promote the circulation of microbial products, thereby stimulating peripheral immune cells to secrete pro-inflammatory cytokines and priming microglia and astrocytes within the CNS [42]. Elevated cytokine levels have also been associated with aberrant activation of other immune products, including IgE immunoglobulins [43], and with alcohol-related organ dysfunction, with cytokines such as TNF-α and IL-6 contributing to alcoholic hepatitis and alcoholic liver disease, respectively [12]. This chronic pro-inflammatory state is also associated with oxidative stress, diminished neural function [44], and possible modulation of neurotransmitter systems relevant to addiction [45]. Taken together, these findings support immune dysregulation as a relevant mechanism underlying the neuropsychological changes observed in AUD [41].

Several studies have reported associations between inflammatory alterations and AUD [23,24,25,26,27,28,29,30]. Peripheral cytokines, including IL-6 and TNF-α [39,46,47], as well as IL-8, IL-10, and IL-12 [24,43], together with inflammatory markers such as CRP [47,48], have been implicated in AUD. Increased peripheral cytokine levels have been associated with clinical manifestations such as insomnia and intestinal problems, psychiatric symptoms including anxiety, depression, and suicide [23,24,25,26,27,28,49], and drinking-related variables such as dependence severity, alcohol withdrawal score, and alcohol craving [39,46,47].

Anxiety and depressive symptoms in AUD were also consistently associated with the reported biomarker profile, linking greater affective symptom burden with heightened inflammatory signalling and reduced neurotrophic support. Specifically, anxiety/depression scores were positively correlated with IL-18 and TNF-α and negatively correlated with BDNF [15,28,41,50,51,52]. These findings suggest that greater affective symptomatology may be associated with a more pronounced proinflammatory milieu and reduced neuroplasticity-related support. Although these correlations do not establish causality, they support the plausibility of an inflammation–affect link as part of the clinical phenotype of AUD [15,28,51]. The inverse association with BDNF further suggests that affective symptoms may co-occur with diminished neurotrophic signalling. This pattern is consistent with the correlational structure reported by Tyler et al. [28], in which greater clinical severity was associated with higher IL-18 and TNF-α and lower BDNF [15,28,50,51,52]. Overall, these results underscore anxiety and depression as clinically relevant dimensions embedded within the broader biological profile of AUD [15,28,50,51,52,53,54].

Cytokine levels may also fluctuate across different stages of chronic alcohol exposure, including active drinking, withdrawal, and abstinence. During active alcohol consumption and early abstinence, pro-inflammatory cytokines such as IL-6, IL-8, TNF-α, and IL-1β tend to be elevated relative to healthy controls, whereas during prolonged abstinence, these levels tend to decrease, suggesting partial recovery of immune function. Mechanistically, alcohol-related increases in intestinal permeability allow bacterial endotoxins, such as LPS, to enter the circulation, activating toll-like receptors (TLR4) and the NF-κB pathway and thereby promoting inflammatory cytokine production [26,42,44]. This process may contribute to systemic inflammation and organ damage, including hepatic and cerebral injury [26,42,44]. A positive association has been reported between cytokine levels and daily alcohol consumption, whereas no significant correlation was observed with alcohol craving [26].

Detoxification studies further indicate that cytokine concentrations may change during treatment. In alcohol-dependent individuals without liver disease, IL-7 remained elevated and later declined, IL-10 increased from low baseline levels, and G-CSF decreased to near-normal values by the end of detoxification [29]. These changes, together with their tendency toward normalization, have been interpreted as indicative of generalized immune disturbance associated with alcohol abuse [29,55,56,57,58,59,60,61].

During abstinence, certain cytokines, particularly IL-6, IL-8, TNF-α, and IL-10, may remain elevated, especially in early abstinence (24–96 h after cessation), and tend to normalize over time [40,46,47]. Elevated IL-8 levels during early withdrawal may reflect acute inflammation [28]. IL-8 was also reported to be higher in abstinent individuals with AUD than in healthy controls and in currently drinking individuals, suggesting ongoing inflammatory activation despite cessation of alcohol use. IL-18 likewise showed a tendency toward higher levels in abstinent participants, which may reflect sustained immune signalling after discontinuation of alcohol consumption. Anti-inflammatory pathways may also be involved, as IL-10 has been linked to AUD through genetic variation and IL-1RA has shown protective effects against alcohol-induced liver injury in preclinical models [28,40,59]. Overall, these findings emphasize the dynamic nature of cytokine regulation in AUD and support the potential utility of cytokines and ILs as biomarkers of disease state and candidate therapeutic targets [14,15,28,41,52].

Although some studies suggest that proinflammatory markers decrease after a few weeks of abstinence, individuals with severe AUD may experience a prolonged proinflammatory state [45,47]. Elevated cytokine levels during abstinence may therefore be linked more closely to the chronicity and severity of AUD than to abstinence itself [14,28]. At the same time, abstinence may confer clinically meaningful immunological benefits by attenuating systemic inflammation and monocyte activation. In this regard, biomarkers of inflammatory activity and gut-barrier dysfunction, including soluble CD14 (sCD14) and IL-6, have been reported to decline after approximately two to three weeks of sustained abstinence [15,23,27,28,41]. Abstinence may also ameliorate co-occurring proinflammatory processes, such as oxidative stress, iron dysregulation, and subclinical hepatic injury, thereby improving clinical trajectories and reducing the likelihood of progression to advanced liver disease [15,23,27,28,41].

This narrative review also acknowledges several limitations, including the small sample sizes, cross-sectional designs, lack of standardized biomarker measurement protocols, and limited representation of diverse populations in the analysed studies. In addition, as a narrative review, the present work was intended to provide a conceptually driven synthesis rather than an exhaustive, protocol-based retrieval of all eligible studies. No formal standardized appraisal of study quality or risk of bias was undertaken, which limits quantitative evaluation of the findings. These characteristics warrant cautious interpretation and highlight the need for future systematic reviews and meta-analyses.

Despite these limitations, this review provides an integrative perspective on the relationship between alcohol use, immune dysregulation, and neurobiology. By synthesizing evidence across clinically relevant phases of AUD, including active drinking, withdrawal, and abstinence, it highlights the dynamic nature of cytokine regulation and the potential translational value of inflammatory and neurotrophic biomarkers. In particular, cytokines such as IL-6, IL-8, TNF-α, and IL-1β may be relevant to biomarker-guided diagnostic strategies, personalized treatment approaches, and relapse prevention.

Overall, the available evidence reinforces the central role of immune dysregulation in the pathophysiology and clinical expression of AUD. Fluctuations in inflammatory markers such as IL-6, IL-8, TNF-α, and IL-1β appear to relate to clinically relevant dimensions of AUD, including symptom severity, affective burden, detoxification course, and relapse vulnerability; however, their prognostic utility remains preliminary and should be interpreted cautiously until confirmed in longitudinal studies.

### 4.1. Clinical Implications

While the available literature points to a possible role of inflammatory pathways in AUD, the translation of these findings into clinical practice remains uncertain. Any discussion of anti-inflammatory or immunomodulatory interventions should therefore be framed cautiously since the current evidence is insufficient to support these strategies as established therapeutic options in AUD. At this stage, such approaches should be considered investigational and mainly relevant for future clinical research.

Clinical implications emerging from this narrative synthesis support a more structured and multidimensional approach to the management of alcohol detoxification and its associated complications. In particular, clinical practice should incorporate an objective protocol for evaluating detoxification outcomes given that a key limitation identified in the evidence base was the absence of objective clinical documentation of treatment response. Implementing standardized instruments such as the Clinical Institute Withdrawal Assessment for Alcohol Scale (CIWA-Ar) would strengthen the clinical characterization of withdrawal severity and detoxification progress and would provide a consistent framework for monitoring clinical response across different settings. In addition, beyond the expected remission of alcohol-induced depressive symptoms, the evidence supports considering assessment of immune status in alcohol-dependent individuals as part of clinical care, with the explicit aim of preventing subsequent complications during and after detoxification.

The reported correlations between AUD severity, anxiety/depression, and biomarkers (IL-18, TNF-α, and BDNF) further argue for an integrated clinical perspective in which inflammatory activity, neuroplasticity-related signalling, and psychological health are considered jointly rather than in isolation. For this purpose, Balan et al. [26] highlights IL-1β as a promising biomarker for assessing risk of AUD, as indicated by scores on the AUDIT and AUDIT-C screening instruments. Across predictive models, IL-1β emerged as the strongest and most consistent biomarker for AUDIT and AUDIT-C outcomes. In linear regression analyses, IL-1β accounted for up to 51% of the variance in AUDIT scores and 38% of the variance in AUDIT-C scores [26].

From a diagnostic perspective, receiver operating characteristic (ROC) analyses indicated that IL-1β has good discriminative performance for identifying individuals with AUDIT scores ≥ 6 (AUC = 0.81) and excellent discrimination for AUDIT-C scores (AUC = 0.94), suggesting potential utility for detecting risky alcohol consumption patterns. Moderated multiple regression (MMR) analyses further supported the independent predictive value of IL-1β. IL-1β predicted AUDIT and AUDIT-C scores without significant interaction effects involving other immunological mediators, indicating that its association with alcohol-risk indices is not contingent on the levels of the other biomarkers examined [26].

Clinically, these findings may suggest that IL-1β may function as an early marker of AUD risk, including among individuals who do not yet meet full diagnostic criteria for AUD. This could facilitate earlier identification of at-risk individuals and enable more tailored preventive or therapeutic interventions. Overall, the study of Balan et al. [26] underscores the potential of IL-1β as a clinically relevant biomarker for assessing AUD risk and its link to inflammatory and immune dysfunction pathways. Nevertheless, the authors emphasize the need to confirm these results in larger samples and in clinically well-characterized cohorts [26].

Within this framework, biomarker findings are clinically relevant because they point to a biological signature that covaries with clinical severity and affective symptom burden. This supports the clinical rationale for viewing biomarkers as potential tools to confirm diagnosis, define current stage, facilitate earlier identification, and complement standard clinical assessments in monitoring the course and outcome of detoxification therapy. Consistent with the longitudinal priorities emphasized in the literature, biomarker monitoring is also positioned as potentially useful for characterizing trajectories during abstinence and for identifying patients at early stages who may be at higher risk of progression or poorer prognosis.

From a management standpoint, the recommendations organized by clinical domains emphasize inflammation as a relevant treatment target in severe presentations and in the presence of persistent inflammatory activation. In this context, anti-inflammatory strategies may warrant investigation, including immunomodulatory medications—such as IL-1 pathway inhibitors or TNF-α antagonists—in individuals with severe AUD and evidence of sustained inflammation. In parallel, naturally derived compounds with reported anti-inflammatory properties (e.g., omega-3 fatty acids, curcumin, or polyphenols) could be explored as adjunctive approaches. These approaches can be complemented by lifestyle modifications, including promotion of a balanced diet rich in anti-inflammatory foods and encouragement of regular physical activity. Given the proposed contribution of gut dysbiosis to systemic inflammation, addressing gut health through probiotics or dietary interventions such as high-fiber diets is also included within the recommended clinical domain focused on inflammation.

The clinical implications also extend to interventions aimed at enhancing neuroplasticity, particularly in the context of lower BDNF levels and comorbid depression/anxiety. Pharmacological avenues proposed within the recommendations include investigating medications that may enhance BDNF, such as antidepressants (e.g., Selective Serotonin Reuptake Inhibitors: SSRIs) or ketamine in individuals with AUD and comorbid affective symptoms, as well as exploring GLP-1 receptor agonists. Non-pharmacological strategies are also emphasized, including mindfulness-based practices such as meditation and yoga and the use of cognitive-behavioural therapy (CBT) to address anxiety, depression, and alcohol-related behaviours. Exercise is highlighted as a central component of this neuroplasticity-oriented domain, with recommendations to incorporate aerobic activity and/or resistance training as part of treatment planning.

Considering the frequent co-occurrence of anxiety and depression in AUD and their associations with the described biomarker profile, mental health assessment and management should be positioned as an integral component of care, not an adjunct. The recommendations therefore emphasize integrated, dual-diagnosis treatment that targets both AUD and co-occurring mental health disorders, alongside the use of psychiatric medications such as SSRIs or SNRIs when indicated. Psychotherapeutic approaches are also foregrounded, including trauma-focused therapy in the presence of early-life stress or PTSD, and motivational interviewing to support engagement and address ambivalence regarding recovery. In addition, participation in peer support groups—such as Alcoholics Anonymous or SMART Recovery—is recommended to reduce isolation and provide ongoing emotional support.

In terms of implementation and follow-up, the recommendations underscore the potential role of systematic biomarker monitoring to support clinical decision-making. This includes regular testing of inflammatory markers (IL-18 and TNF-α) and neuroplasticity-related biomarkers (BDNF) during treatment, with the goal of evaluating progress over time. Within this approach, biomarker-guided therapy is proposed, whereby clinical emphasis is tailored according to biomarker levels—for example, prioritizing anti-inflammatory strategies when IL-18 or TNF-α are elevated, and emphasizing neuroplasticity-oriented strategies in the context of low BDNF and prominent affective symptoms.

Finally, sustained abstinence support is identified as a critical component of management, particularly for individuals with severe AUD who may require more intensive early-phase stabilization. The recommendations include consideration of residential or inpatient programs during early abstinence to provide structured care and therapy, as well as relapse prevention strategies that combine pharmacotherapy to reduce cravings with ongoing therapy and relapse prevention planning. Across all domains, a holistic, personalized, and multidisciplinary model of care is recommended that involves collaboration among addiction specialists, psychologist, psychiatrists, nutritionists, and physical therapists. Patient education is also highlighted as an essential component, with an emphasis on explaining the connections between alcohol use, inflammation, and mental health to support informed engagement with treatment and recovery planning. Table 3 summarizes the clinical implications and potential applications of cytokine and interleukin biomarkers in AUD. This table provides a comprehensive overview of how specific biomarkers can be utilized to enhance diagnostic precision, monitor disease progression, and inform personalized potential future therapeutic avenues. By highlighting the translational relevance of these biomarkers, the table serves as a practical guide for integrating immunological insights into clinical practice and developing targeted interventions for AUD management. Nevertheless, caution is warranted when interpreting these findings. Much of the available literature is cross-sectional in nature, and results are heterogeneous across studies. Moreover, several factors, such as liver disease, smoking, psychiatric comorbidities, and withdrawal stage, may affect circulating cytokine levels and complicate the interpretation of their relationship with AUD.

### 4.2. Future Lines of Research

Before outlining future lines of research, it should be noted that the available evidence on inflammatory alterations in AUD remains limited by the predominance of observational, largely cross-sectional studies, as well as by substantial methodological and clinical heterogeneity across samples. Accordingly, any potential therapeutic implications derived from these findings should be interpreted with caution. At present, inflammatory pathways should be primarily regarded as promising areas for further investigation rather than as a basis for established treatment strategies in AUD.

Based on analysed studies, future research should adopt longer follow-up periods and longitudinal designs to clarify the long-term effects of alcohol detoxification on cytokine concentrations and the recovery of immune system function. In addition to examining post-detoxification change, longitudinal work should specifically investigate temporal trajectories in biomarker levels—such as CD163, sCD14, and IL-6—particularly following alcohol abstinence, to better characterize their evolution over time and their potential roles in the progression of alcohol-related conditions. These approaches would also help address a key limitation noted in the current review in reference to the absence of objective clinical evidence documenting detoxification treatment outcomes.

To strengthen clinical characterization, future studies should incorporate standardized clinical evaluations of detoxification outcomes, for example, through implementation of protocols such as the Clinical Institute Withdrawal Assessment for Alcohol Scale (CIWA-Ar), thereby enabling immune measures to be interpreted alongside objectively documented clinical course.

Improving the representativeness and clinical scope of study populations is also a priority. Future research should expand to include more diverse patient groups, including alcohol-dependent individuals with liver disease or other co-existing conditions in order to capture the broader impact of alcohol abuse on immune function. Targeted analyses in specific cohorts with alcohol-induced severe hepatic and pancreatic disease are warranted to identify the stages at which cytokines such as IL-6 and IL-17A may function as disease-specific biomarkers. Complementarily, including individuals who are actively consuming alcohol would enable more precise characterization of the direct effects of alcohol exposure on cytokine concentrations and their relationship with comorbidities. Broader sampling across drinking phenotypes—including individuals with milder patterns of alcohol use or moderate drinking—would allow for comparisons of biomarker profiles and help clarify their relevance across different levels of alcohol consumption. Future studies should also increase efforts to recruit women, explicitly considering factors such as the menopausal transition and hormonal levels (e.g., estrogen and luteinizing hormone) that may influence cytokine concentrations. Furthermore, larger sample sizes are required to improve statistical validity and enhance the generalizability of findings. In addition, assessing psychiatric patients without a history of substance use disorders would help distinguish cytokine alterations attributable to AUD from those associated with other psychiatric conditions.

Beyond population-level refinements, future work should broaden the immunological scope of measurement. Studies incorporating additional immune parameters, together with longer follow-up periods and varied alcohol-dependent patient groups (including those with established liver disease), are needed to further elucidate pathophysiology. Within this framework, research should evaluate the potential utility of IL-7, IL-10, and G-CSF as biomarkers for monitoring progress and outcomes during detoxification therapy. More generally, biomarkers may be useful for confirming diagnosis, defining the current stage of AUD, and supporting early identification of patients. IL-6 elevation in individuals with AUD is also proposed as a potential risk marker for adverse medium-term outcomes even in the absence of advanced liver disease, and future studies should determine whether biomarker panels can identify early-stage AUD patients at increased risk of mortality or progression to severe liver disease.

Mechanistic investigation remains essential to explain observed biomarker patterns. Future research should examine interactions among cytokines—such as IL-7, IL-10, G-CSF, and other mediators—to improve our understanding of immune modulation in alcohol dependence. Future research should further explore the mechanisms through which alcohol abuse leads to immune system disorders, including cytokine pathway dysregulation and immune cell dysfunction. Given the complexity of inflammatory signalling, developing integrated models that synthesize information across multiple inflammatory signals may better capture cytokine interaction patterns in alcohol addiction and its comorbidities. Additional mechanistic priorities include elucidating the links between monocyte activation, systemic inflammation, and metabolic disturbances in AUD even in the absence of advanced liver disease. At the genomic and molecular level, studies should investigate mechanisms underlying cytokine alterations during alcohol detoxification, including the role of transcription factors such as STAT3. The relationship between alcohol-induced autoimmune reactions and cytokine production—particularly IL-7—also warrants further study to clarify its proposed relevance for liver function and immune system regulation.

Interventional and comparative research directions are likewise proposed. Future studies should examine the specific effects of pharmaceutical treatments used during detoxification, including diazepam and vitamin supplementation, on cytokine levels and immune recovery. Comparative studies evaluating cytokine alterations across different detoxification protocols are needed to identify the most effective treatment approaches. In addition, research should assess how targeted interventions—such as treatment of hepatitis C virus infection, management of metabolic abnormalities, and sustained abstinence—affect biomarker levels and clinical outcomes. Given the clinical complexity of AUD, further work should explore interactions between AUD and comorbidities such as HCV infection, metabolic syndrome, and cardiovascular disease, with the aim of informing integrated therapeutic strategies.

Furthermore, future research should delve deeper into the relationship between biomarkers and comorbid conditions, such as hepatic and pancreatic diseases, which are frequently associated with AUD. While current studies have identified associations between elevated cytokine levels—such as IL-6 and IL-17A—and liver and pancreatic dysfunction, further investigation is needed to elucidate the underlying mechanisms driving these interactions. Understanding how specific biomarkers contribute to the progression of these comorbidities could provide a foundation for the development of targeted prevention and potential future therapeutic avenues. For instance, identifying early-stage biomarkers of liver or pancreatic disease in AUD patients may enable timely interventions to mitigate disease progression. Additionally, exploring the role of anti-inflammatory cytokines, such as IL-10, in protecting against organ damage could inform the design of novel therapeutic approaches aimed at restoring immune balance and reducing the burden of comorbid conditions in AUD populations.

Finally, future research should translate these immunological insights into preventive and clinically useful applications. Developing strategies to assess and improve the immune status of alcohol-dependent individuals may help prevent complications during and after detoxification. Within detoxification and follow-up care, immunological parameters may serve as biological markers and diagnostic tools to assess the course and outcome of therapy. More broadly, the framing of cytokines as potential biomarkers of psychiatric disorders provides an additional rationale for integrating immune profiling into research that addresses AUD and its comorbidities.

In sum, to advance the understanding of immune dysregulation in AUD, future research should adopt more targeted and interdisciplinary approaches. Specifically, the following strategies are proposed:

– Longitudinal study designs: Conduct extended follow-up studies to monitor the temporal evolution of cytokine and interleukin levels during different stages of AUD, including active drinking, withdrawal, and sustained abstinence. These studies should incorporate repeated biomarker assessments at multiple time points to capture dynamic changes in immune profiles.
– Advanced methodologies: Utilize cutting-edge techniques such as single-cell RNA sequencing and proteomics to investigate cytokine interactions and immune cell-specific responses. These methods can provide deeper insights into the molecular mechanisms driving immune dysregulation in AUD.
– Interdisciplinary collaborations: Establish partnerships between psychologist, psychiatrists, addiction specialists, immunologists, neuroscientists, and bioinformaticians to develop integrated models of AUD pathophysiology. Collaborative efforts can facilitate the synthesis of data across immune, neurobiological, and behavioural domains, enabling a more holistic understanding of the disorder.
– Clinical trials for biomarker validation: Design randomized controlled trials to evaluate the utility of cytokines and ILs as biomarkers for AUD diagnosis, treatment response, and relapse prediction. These trials should include diverse patient populations, such as individuals with varying levels of alcohol consumption and comorbid conditions.
– Development of targeted interventions: Investigate whether immunomodulatory approaches, such as cytokine-targeted or other anti-inflammatory interventions, may have a role in AUD-related inflammatory dysregulation. At present, the available evidence is limited, and these strategies should be considered exploratory rather than clinically established therapeutic options.
– Global and population-based studies: Expand research to include diverse populations across different geographic regions and cultural contexts. This approach will help identify population-specific immune signatures and inform tailored interventions.

By implementing these strategies, future research can address existing gaps in the literature, enhance the clinical applicability of findings, and contribute to the development of innovative approaches for the prevention and treatment of AUD. While these lines of research appear promising, they should be interpreted cautiously in light of the current limitations of the available evidence.

## 5. Conclusions

This narrative review highlights the critical role of cytokine and interleukin networks in the pathophysiology of AUD, emphasizing their involvement in immune dysregulation, systemic inflammation, and neuroimmune signalling. The scientific evidence suggests that chronic alcohol consumption disrupts immune homeostasis, leading to elevated levels of pro-inflammatory markers such as IL-6, TNF-α, and IL-8, alongside alterations in anti-inflammatory mediators like IL-10. These immune changes are closely linked to the clinical severity of AUD, as well as comorbid affective symptoms such as anxiety and depression.

The review underscores the dynamic nature of cytokine regulation across different stages of AUD, including active drinking, withdrawal, and abstinence. While abstinence appears to promote partial normalization of immune function, individuals with severe AUD may experience prolonged inflammation even after cessation of alcohol use. This highlights the relevance of further investigating the relationship between inflammation, neuroplasticity deficits, and clinical manifestations in AUD. Although these findings may eventually inform more personalized approaches, the therapeutic implications of targeting inflammatory pathways remain preliminary.

The findings also point to the potential utility of cytokines, such as IL-1β, IL-6, and IL-18, as biomarkers for assessing AUD severity, monitoring treatment progress, and predicting clinical outcomes. These biomarkers could serve as valuable tools for early identification of at-risk individuals and for guiding personalized interventions.

Despite the promising insights, significant research gaps remain. Future studies should focus on longitudinal designs, larger and more diverse cohorts, and standardized clinical assessments to better understand the long-term trajectories of immune recovery and the interplay between cytokine alterations, neurobiology, and clinical phenotypes in AUD. Additionally, mechanistic investigations are needed to elucidate the pathways through which alcohol-induced immune dysregulation contributes to neuroinflammation and organ damage. Although inflammatory pathways may be of interest as potential therapeutic targets, current evidence is still insufficient to support anti-inflammatory interventions as established treatments for AUD.

To sum up, this review emphasizes the potential relevance of immune profiling in improving the understanding of AUD pathophysiology and in informing future research on patient stratification and treatment monitoring. However, its routine use in clinical management and the therapeutic targeting of inflammatory pathways still require stronger clinical evidence.

## Figures and Tables

**Table 1:** Overview of Inflammatory Markers Associated with Alcohol Use Disorder (AUD).

Category	Marker	Description
Pro-inflammatory cytokines	IL-6	Associated with pro- and anti-inflammatory activities; linked to the pathology of alcohol-induced liver disease.
Pro-inflammatory cytokines	IL-7	Contributes to the inflammatory response.
Pro-inflammatory cytokines	IL-8	Related to inflammatory processes and tissue injury.
Pro-inflammatory cytokines	TNF-α	Implicated in inflammation and liver damage in AUD.
Anti-inflammatory cytokines	IL-10	Has anti-inflammatory properties; concentrations may fluctuate depending on the stage of AUD.
Other markers	IL-1β	Related to inflammation and neuronal injury.
Other markers	IL-12	Associated with immune responses in patients with AUD.
Other markers	TGF-β1	Linked to inflammatory processes and immunological alterations.

**Note:** Table developed by authors from the data reported in the analysed studies. These markers show altered concentrations in patients with AUD, particularly during phases of active drinking and abstinence; fluctuations reflect the complexity of immune responses in AUD and their contribution to systemic inflammation and organ damage.

**Table 2:** Main limitations of the most relevant analysed studies.

Study	Main Limitations
Nikou et al. [29]	– Absence of objective clinical evidence: The study lacks objective clinical evidence for the detoxification treatment outcome, such as the implementation of the Clinical Institute Withdrawal Assessment of Alcohol Scale (CIWA-Ar). – Short follow-up period: The study only covers a 4–5-week detoxification period, which may not provide a comprehensive understanding of long-term immune system recovery or changes in cytokine levels. – Limited patient group: The study focuses solely on alcohol-dependent individuals without liver disease, excluding those with established liver disease or other substance abuse, which limits the generalizability of the findings. – No assessment of immune status for prevention: The study does not assess the immune status of alcohol-dependent individuals for the prevention of further complications. – Unclear influence of treatment: It is uncertain whether the observed alterations in cytokine concentrations are solely due to alcohol withdrawal or influenced by the supportive pharmaceutical treatment (diazepam and vitamins). – No data on interaction between cytokines: The study lacks complementary data on the interaction between IL-7 and other cytokines in the context of alcoholism.
García-Marchena et al. [23]	– Small number of female participants: The study cohort included a limited number of female participants, which may affect the generalizability of the findings. Future studies should aim to recruit more female participants and consider factors such as hormonal changes during menopause. – Exclusion of active alcohol consumers: The study only included abstinent AUD patients, which limits the ability to analyse the direct effects of active alcohol consumption on cytokine concentrations. – Small sample size: The study was conducted with a relatively small sample size, which may limit the statistical power and generalizability of the findings. Larger cohorts are needed to validate the results. – Lack of psychiatric controls: The study did not include psychiatric patients without a history of substance use disorders, which could provide a better understanding of the specific impact of AUD on cytokine levels. – Limited focus on severe liver/pancreatic disease: The study was not specifically designed to analyse patients with severe alcohol-related liver or pancreatic diseases, which may limit the applicability of the findings to these populations. – Complexity of cytokine interactions: The study acknowledges the complexity of inflammatory signalling and the need for further research to fully understand the role of cytokines in the aetiology of AUD and its associated comorbidities.
García-Calvo et al. [27]	– Lack of a healthy control group: The study did not include healthy participants for comparison, which limits the ability to interpret the findings relative to the general population or to moderate drinkers. – Specific study population: Participants were patients with severe AUD, characterized by a high median alcohol intake (142 g/day). Consequently, the results may not be generalizable to individuals with milder patterns of alcohol use. – Single time-point biomarker assessment: Biomarkers (CD163, sCD14, IL-6) were only measured once, two days after admission. Because levels of these markers may decrease with longer alcohol abstinence, temporal changes could not be evaluated. – Absence of longitudinal follow-up: The study did not assess long-term trajectories of biomarker levels or their prognostic value for predicting mortality risk or disease progression.
Tyler et al. [28]	– Small sample size: Each group (HC: Healthy controls; CD: Nontreatment seeking; and AB: Abstinent treatment-seeking) includes a small number of participants (approximately 10 per group), reducing statistical power and increasing the risk of biased or unrepresentative results. This limits the generalizability of the findings. – Premature data collection halt: Data collection was stopped due to the COVID-19 pandemic, preventing the study from reaching its intended sample size. – Preexisting differences between AUD groups: The AUD groups (CD and AB) differ in terms of AUD severity, alcohol consumption levels, and anxiety/depression measures prior to abstinence. These differences complicate the interpretation of results, making it difficult to determine whether observed differences are due to abstinence or preexisting factors. – Lack of pre-abstinence data for the AB group: Biomarkers were not measured before abstinence in the AB group, limiting the ability to fully understand changes during the abstinence period. – Reliance on self-reported data: Alcohol consumption measures rely on participants’ self-reports, which may introduce bias or inaccuracies. Although breathalyser tests were conducted, there is no biological confirmation of abstinence, such as phosphatidyl ethanol (PEth) testing. – Incomplete biomarker analysis: Some important biomarkers, such as IL-1β, were not included in the analysis, leaving an incomplete picture of IL-1 receptor signalling pathways. – Use of aggregate values: Aggregate values for pro- and anti-inflammatory biomarkers were derived through principal component analysis (PCA) and should be interpreted cautiously as some biomarkers can have both pro- or anti-inflammatory effects.
Balan et al. [26]	– Cross-sectional design: The study’s cross-sectional nature prevents distinguishing between the inflammatory effects of past alcohol exposure and those related to current drinking patterns. – Small sample size: The study analysed data from only 28 participants, which limits the generalizability of the findings and reduces statistical power, particularly for subgroup analyses (e.g., sex-based differences or interaction effects). – Focus on AUDIT Scores: The study relied on AUDIT and AUDIT-C scores to stratify alcohol-related risk, rather than using comprehensive diagnostic assessments for DSM-5-defined AUD. This limits the ability to directly associate immune biomarkers with formal AUD diagnoses and severity subtypes. – Potential confounding factors: Although efforts were made to minimize confounding variables (e.g., smoking, autoimmune diseases, depression), it is challenging to fully isolate the effects of alcohol use from other environmental and lifestyle factors, such as diet, stress, or other substance exposures. – Limited scope of biomarkers: The study primarily focused on proinflammatory cytokines and chemokines, without including anti-inflammatory markers (e.g., IL-1RA, IL-10) that could provide a more comprehensive understanding of immune dysregulation in AUD. – Exploratory nature of PCA: The principal component analysis (PCA) was conducted on a small sample size, making the findings exploratory and potentially less reproducible in larger cohorts. – Lack of leukocyte subtype analysis: The study did not assess specific leukocyte subtypes, which could provide more detailed insights into immune cell function and contributions to the observed immune dysregulation. – Uncertainty in translational potential: While the findings suggest IL-1β as a promising biomarker for AUD risk, further research in larger, diagnostically defined cohorts is needed to validate its clinical applicability.
Bramness et al. [25]	– Sample size: The study was conducted in a relatively small cohort of patients with AUD, which may have resulted in insufficient statistical power to detect significant differences in risk factors for suicide attempts. – Selection bias: Participant selection was restricted, as individuals with severe somatic illness, psychosis, cognitive impairment, or inability to speak a Scandinavian language were not included. This may have limited the generalizability of the findings. – Assessment of suicide attempts: Suicide attempts were assessed as a dichotomous self-reported variable, without accounting for time since the most recent attempt, the method used (violent vs. non-violent; impulsive vs. non-impulsive), or the number of attempts. – Comorbidities and confounding: The high comorbidity among AUD, major depressive disorder (MDD), and somatic conditions may complicate interpretation of the results and hinder identification of differences in cytokine levels. – Methodological constraints: The cross-sectional design precludes causal inference regarding the relationship between IL-6 levels and suicide attempts. In addition, the limited sample size constrained the number of variables that could be included in the multivariable analysis. – Overall cytokine elevation in AUD: The generalized elevation of cytokines in patients with AUD may have masked differences that might otherwise have been detectable in cytokine levels associated with suicide attempts.
Grodin et al. [24]	– Sample size: While the overall sample size was relatively large, some groups, particularly the clinical insomnia group, had modest sample sizes. This limits the generalizability of the findings, especially for interactions between AUD severity and insomnia symptoms. – Cross-sectional design: The study is based on a cross-sectional analysis, meaning it cannot determine the temporal or causal relationships between alcohol use, insomnia symptoms, and inflammation. – Insomnia measurement: the study uses the Insomnia Severity Index (ISI), a self-reported measure. While the ISI is validated, it does not provide the detailed data that could be obtained through polysomnography, the gold standard for assessing sleep disorders. – COVID-19 Context: Data were collected during the COVID-19 pandemic, which may have introduced biases related to recent exposure to the virus or vaccination. Although covariates were included to mitigate these effects, they were not significant. – Diurnal variations in inflammatory markers: Blood samples were not collected at the same time of day for all participants, which could have influenced inflammatory marker levels due to circadian variations. While the time of collection was included as a covariate, it was not significant.
Papantoniou et al. [30]	– Small Sample Size: The study had a relatively small sample size, which may limit the generalizability of the findings. – Lack of skin biopsies: Skin biopsies were not performed, which may have led to undetected cases of small-fiber neuropathy among the patients. – Timing of cytokine measurements: Serum cytokine concentrations were measured at the very early stage of the detoxification program. This means that potential changes in cytokine levels during the later stages of detoxification were not captured or analysed.

**Note:** Table was prepared by the authors to summarize the main limitations of the most relevant analysed studies.

**Table 3:** Clinical implications and potential applications of cytokine and interleukin biomarkers in Alcohol Use Disorder (AUD).

Clinical Domain	Biomarker Signal(s)	Practical Application in Clinical Practice	Example Implementation	Clinical Value/Expected Benefit
Biomarker-guided diagnosis and risk stratification	Elevated IL-6, IL-8, TNF-α, IL-1β	Support identification of individuals at risk for AUD or in early-stage AUD as an adjunct to standard clinical assessment.	Add cytokine panel to routine laboratory work-up in patients with suspected AUD (alongside clinical interview and screening tools).	Earlier detection, improved phenotyping, and more objective risk stratification.
Treatment monitoring across detoxification and abstinence	Longitudinal trajectories of IL-6, IL-10; persistent elevation of IL-8	Track biological response to treatment and flag persistent immune activation during abstinence.	Serial measurements during detoxification and follow-up visits; persistent IL-8 elevation prompts closer monitoring and targeted supportive care.	More individualized follow-up intensity; potential early warning for ongoing inflammation and vulnerability.
Personalized pharmacological adjuncts	High pro-inflammatory profile (e.g., TNF-α, IL-18; IL-1β)	Potential future therapeutic avenues for immunomodulatory strategies as adjuncts to standard AUD care (investigational/off-label depending on context).	Consider evaluation of TNF-α inhibitors or IL-1 receptor antagonists in clinical trials on severe AUD with marked inflammation.	Potential reduction in systemic inflammation and improvement in symptom burden or medical comorbidity (requires rigorous validation).
Personalized lifestyle and behavioral interventions	Elevated inflammatory markers; gut-related inflammatory signatures	Implement anti-inflammatory lifestyle interventions to complement standard AUD treatment.	Nutritional counseling emphasizing omega-3 fatty acids, polyphenols, and high-fiber diets; structured physical activity plans.	Reduced inflammatory tone, improved metabolic and gut health, and broader supportive effects on recovery.
Mental health integration within AUD care	Associations of IL-18 and TNF-α with anxiety/depression symptoms	Integrate psychiatric evaluation and treatment in patients with inflammatory activation and affective symptoms.	Combined care pathways: evidence-based psychotherapy (e.g., CBT, mindfulness-based interventions) plus optimized AUD treatment; consider inflammation-informed monitoring.	Improved management of comorbidity, potentially better adherence and outcomes via integrated biopsychosocial care.
Relapse prevention and stepped-care intensity	Persistent inflammatory activation; reduced neuroplasticity-related markers (e.g., BDNF)	Identify patients at elevated relapse risk to escalate relapse-prevention intensity.	High-risk profiles prompt stepped-up care: residential programs, pharmacotherapy, and structured continuing care.	More targeted allocation of intensive resources; potentially improved relapse prevention.
Early preventive intervention before severe AUD	Elevated IL-1β in individuals with high AUDIT scores	Offer preventive support to high-risk individuals prior to severe AUD onset.	Use biomarker + AUDIT-informed triage to deliver motivational interviewing, brief interventions, and peer-support referral.	Earlier intervention window; potential reduction in progression to severe AUD.
Multidisciplinary care model development	Multi-marker inflammatory profiles (cytokines/ILs; neuroplasticity markers)	Coordinate integrated care addressing inflammation, neurobiology, and mental health.	Team-based management involving addiction specialists, psychologists, psychiatrists, immunology-informed input, nutrition, and physical therapy.	Holistic care, improved coordination, and more comprehensive management of comorbidities.
Patient education and engagement	Biomarker-informed explanation of immune effects of alcohol	Use biomarker results to enhance patient understanding and adherence.	Clinician-led education linking alcohol use to immune dysregulation and mental health; shared decision-making around treatment plan.	Greater engagement, adherence, and self-efficacy through personalized feedback.

**Note:** Table developed from the data reported in the analysed studies.

## Data Availability

No new data were created or analyzed in this study.
